# Study on the Estimation of Forest Volume Based on Multi-Source Data

**DOI:** 10.3390/s21237796

**Published:** 2021-11-23

**Authors:** Tao Hu, Yuman Sun, Weiwei Jia, Dandan Li, Maosheng Zou, Mengku Zhang

**Affiliations:** School of Forestry, Northeast Forestry University, Harbin 150040, China; 2019115037@nefu.edu.cn (T.H.); symsfs@nefu.edu.cn (Y.S.); lidand@nefu.edu.cn (D.L.); 18213470376@nefu.edu.cn (M.Z.); mkzero@nefu.edu.cn (M.Z.)

**Keywords:** forest volume, multi-source remote sensing factor, ordinary Kriging (OK), random forest (RF), support vector regression (SVR), artificial neural network (ANN)

## Abstract

We performed a comparative analysis of the prediction accuracy of machine learning methods and ordinary Kriging (OK) hybrid methods for forest volume models based on multi-source remote sensing data combined with ground survey data. Taking *Larix olgensis*, *Pinus koraiensis*, and *Pinus sylvestris* plantations in Mengjiagang forest farms as the research object, based on the Chinese Academy of Forestry LiDAR, charge-coupled device, and hyperspectral (CAF-LiTCHy) integrated system, we extracted the visible vegetation index, texture features, terrain factors, and point cloud feature variables, respectively. Random forest (RF), support vector regression (SVR), and an artificial neural network (ANN) were used to estimate forest volume. In the small-scale space, the estimation of sample plot volume is influenced by the surrounding environment as well as the neighboring observed data. Based on the residuals of these three machine learning models, OK interpolation was applied to construct new hybrid forest volume estimation models called random forest Kriging (RFK), support vector machines for regression Kriging (SVRK), and artificial neural network Kriging (ANNK). The six estimation models of forest volume were tested using the leave-one-out (Loo) cross-validation method. The prediction accuracies of these six models are better, with $R_{Loo}^{2}$ values above 0.6, and the prediction accuracy values of the hybrid models are all improved to different extents. Among the six models, the RFK hybrid model had the best prediction effect, with an $R_{Loo}^{2}$ reaching 0.915. Therefore, the machine learning method based on multi-source remote sensing factors is useful for forest volume estimation; in particular, the hybrid model constructed by combining machine learning and the OK method greatly improved the accuracy of forest volume estimation, which, thus, provides a fast and effective method for the remote sensing inversion estimation of forest volume and facilitates the management of forest resources.

## 1. Introduction

As an important part of the global ecosystem, the forest landscape plays an important role in maintaining the global carbon emission balance and curbing global warming, in which context forest volume is one of the important indicators [1,2]. In order to actively respond to climate change, take the path of green and low-carbon development, and achieve sustainable development, China has proposed to achieve the goal of carbon neutrality and zero emissions by 2060, and increasing forest volume is an important means to achieve this goal [3,4,5,6]. There are many methods for investigating the volume of the forest inventory in forestry surveys. A traditional forest inventory is usually obtained using the first- and second-class survey data of national forest resources. This method is long and time-consuming and, thus, is not conducive to a quick assessment of the forest volume of a region. We must, thus, improve the existing research methods and introduce new technical means [7]. With the maturity of remote sensing, synthetic aperture radar, and LiDAR technologies, it is possible to estimate forest volume at a large scale and with temporal efficiency [8,9,10,11,12,13].

In the field of remote sensing, vegetation indices provide a simple and effective measure of surface conditions, which can reflect vegetation vitality and information [14,15]. At present, the most commonly used vegetation indices are constructed from a combination of visible and near-infrared bands, and the vegetation indices constructed solely based on the visible band are rarely applied in forestry. However, visible vegetation indices based on unmanned aerial vehicle (UAV) images have been widely used in crop recognition [16], biomass estimation [17,18], and information extraction [19,20]. In general, most remote sensing image data obtained by UAVs only has visible light bands (RGB) and no near-infrared, because the acquisition cost is low [21]. Studies have shown that there is a correlation between a visible vegetation index (includes visible light bands) and a vegetation index (includes common bands such as visible and near-infrared bands), and the visible vegetation index can also sometimes replace the near-infrared vegetation index in inversion research [22,23]. In addition to the vegetation index, texture features also have an impact on forest volume inversion. At present, vegetation texture features are mainly used in vegetation coverage and land use change research, and forestry remote sensing images are mainly used to estimate forest biomass and leaf area index [24]. Chubey et al. performed a plot-oriented analysis of texture features to extract forest age and tree height parameters from high-resolution remote sensing images [25]. In recent years, texture features have been used to estimate forest volume, which can effectively solve the problem of “same thing, different spectrum” in remote sensing images and improve the accuracy of image information extraction by making full use of texture information [26,27].

As an active remote sensing technology, LiDAR has a strong penetration ability, independence from shadows, and a strong anti-interference ability. It can overcome problems such as the easy saturation of traditional optical remote sensing [28,29]. An airborne laser LiDAR scanning system is composed of a laser altimeter, a GNNS positioning device, an inertial guidance instrument, and a high-resolution digital camera for the synchronous measurement of a target [30,31,32]. In the 1980s, experts and scholars from many countries began to study the application of LiDAR technology in forestry surveys [33]. After years of research and practice, much has been achieved in the estimation of forest volume via LiDAR technology, providing valuable experience for the application of LiDAR technology in this area [29]. Næsset and Økland used LiDAR to extract point cloud height variables and density variables to fit prediction models and grouped them according to different stand ages and stand conditions to estimate stand volume in southeastern Norwegian forests by building log-transformed multiple linear regression models [34]. Bottalico et al. used coniferous plantation forests in the Italian Mediterranean and established several stand feature inversion models by extracting LiDAR feature variables and combining them with actual measurement data from sample plots [30]. Li et al. used different algorithms to estimate stand height based on UAV laser scanning data [35]. Silva et al. estimated the stem biomass of eucalyptus plantations in São Paulo, Brazil, by constructing a linear model using airborne LiDAR data [36]. Most of the above research methods use traditional statistical approaches such as linear and nonlinear regression models and mixed-effect models. However, these methods often need to satisfy certain statistical assumptions in their application [37]. Because forest growth is a complex nonlinear process, affected by many factors such as heredity, climate, stand, and their interactions, empirical models are still faced with the challenges of model selection, variable selection, and parameter convergence [38].

With the development of artificial intelligence technology, machine learning algorithms provide a new method for forest growth and harvest prediction. They have the advantages of making no assumptions about the distribution of input data; being able to deal well with the complex relationship between dependent variables and independent variables; the capacity for the deep mining of valuable information from data, revealing their implicit structure; and the construction of better prediction models. Machine learning has been widely used in forest growth and harvest prediction algorithms, but the application of other kinds of machine learning algorithms is not extensive or in depth [39,40,41,42]. However, whether it employs a parametric or nonparametric model, the above research does not take into account the spatial autocorrelation of forest volume, so a hybrid approach that considers both relevant environmental factors and the effect of spatial autocorrelation is required that has a higher prediction accuracy compared with a single model [43,44]. Wasko et al. conducted a comparative study on different combination methods using ordinary Kriging (OK) interpolation with the global residual of the local model, which helps address the spatial autocorrelation of the model residual [45]. Much of the remote sensing data are derived from one single platform, and for various reasons (e.g., technology, cost, etc.), it is difficult to achieve the simultaneous acquisition of optical remote sensing data and LiDAR data using one platform [28]. As the cost decreases, these sensors are becoming more and more popular, thus providing opportunities to develop multi-sensor systems and a basis for combined LiDAR and optical remote sensing techniques. The research of experts and scholars in some countries has explored the integration of LiDAR and hyperspectral scanners on different platforms. Pang et al. compared the utility of airborne LiDAR and spaceborne hyperspectral data for estimating forest leaf area index; these data were obtained from different platforms and in different time periods, which affects the fusion potential and is, thus, bound to lead to prediction error [28]. Therefore, the development of a multi-sensor integrated airborne remote sensing system for accurate data fusion has become a goal [46,47]. The existing airborne remote sensing systems can integrate multi-dimensional data such as high-resolution CCD images, hyperspectral data, and airborne LiDAR point cloud data, which have great potential applicability in forestry investigations.

Many studies have been completed on estimating forest volume using optical remote sensing images and airborne LiDAR point cloud data, but few have combined the variable factors extracted from these two data sources to estimate the forest volume in a region. In this study, we obtained multi-source remote sensing data for the Mengjiagang forest farm using the CAF-LiTCHy airborne observation integration system, and extracted the visible light vegetation index, texture feature, terrain factor, and laser radar point cloud feature variables. Combining this with the measured data of ground plot volume, we constructed an RF model, an SVR model, and an ANN model, as well as RFK, SVRK, and ANNK hybrid models, based on the residual OK interpolation of the machine learning model. We then used these to estimate the forest volume in the study area, providing an efficient method for forest resource management research.

## 2. Materials and Methods

### 2.1. Overview of the Study Area

**Figure 1:**
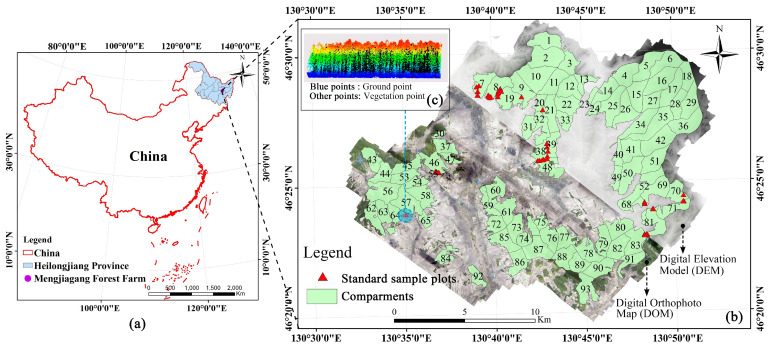
Study area profile and multi-source dataset. The figure shows the specific location of the Mengjiagang forest farm (**b**) in China (**a**). The distribution of ground standard sample plots in forest compartments, the DOM used for extracting vegetation indices, and the DEM for extracting terrain factors are included in (**b**). (**c**) shows the LiDAR point cloud data of a ground standard plot.

This study’s area was the forest area of the Mengjiagang forestry in Jiamusi City, Heilongjiang Province. The forest farm was founded in February 1956. Its geographical coordinates are 130°32′42″–130°52′36″ E and 46°20′16″–46°30′50″ N (as in Figure 1). It exists in a temperate continental monsoon climate, where winter is long, cold, and dry, and summer is short, warm, and humid. The annual ≥10 °C accumulation temperature is about 2547 °C. The average annual precipitation is about 550 mm, and it receives 1955 h of sunshine throughout the year. The frost-free period is about 120 days. Because the weather is cold, the growth period of plants is generally from May to September. The forest farm consists mostly of low hills with gentle slopes (between 10° and 20°), with altitudes between 170 m and 575 m. The soil is dominated by typical dark brown loam. The forest farm mainly manages coniferous plantations of *Larix olgensis*, *Pinus sylvestris*, and *Pinus koraiensis*. Its shrubs and vines include *Acanthopanax senticosus*, *Corylus mandshurica*, etc. Its herbs are primarily *Pteridophyta*, *Convallaria majalis*, *Carex* spp, *Menispermum dauricum*, etc. The Mengjiagang forest farm is rich in forest resources, with a total forest area of 16,274 ha, a total forest volume of 640,000 m^3^, and a forest coverage rate of 80.4%.

### 2.2. Data Acquisition

#### 2.2.1. Ground Standard Land Survey

A field survey was conducted in July 2020 on 52 standard sample plots in the study area of Mengjiagang forest farm, with sample plot areas ranging from 0.06 to 0.2 ha, including 41 *Larix olgensis* plantations, 5 *Pinus koraiensis* plantations, and 6 *Pinus sylvestris* plantations, and a total of 4400 sample trees were investigated. The height, diameter at breast height, crown width, and relative coordinates of each tree were measured in the standard sample plots. The coordinates of the center point and the four corner points of the ground standard sample plots were obtained using real-time kinematic (RTK) differential positioning for more than 30 min at each point, the error for which is generally guaranteed to be within 1 m.

#### 2.2.2. Remote Sensing Data Acquisition

The remote sensing data of this study were acquired via flight scanning Mengjiagang forest area with CAF-LiTCHy, an airborne optical full-spectrum remote sensing system integrated by the China Academy of Forestry Sciences, which comprises four types of ground observation sensors, including LiDAR, a thermal infrared camera, a charge-coupled device (CCD) camera, and a hyperspectral sensor, as well as a high-precision positioning and orientation system (POS) [28]. This system can simultaneously acquire vertical and horizontal structure data, as well as spectral and temperature information, and has the capacity for the remote sensing monitoring of large areas of forest and grass, with a good technical guarantee for the study of forest accumulation.

1. Airborne CCD

Airborne CCD images of the study area were acquired by scanning the Mengjiagang forest farm with the CAF-LiTCHy system, which uses a medium-sized airborne digital camera system (DigiCAM-60) as the CCD sensor. The DigiCAM-60 has 60 megapixels (8956 × 6708), a 1.6 s image repetition rate, and a 16-bit recording depth. The focus lens is 50 mm. The camera has a spatial resolution of 12 cm and a flight altitude of 1000 m [28].

2. Airborne LiDAR

The integrated system LiDAR scanner is a VQ-580II from Riegl, Austria. The VQ-580II laser scanner’s scanning rate is 30–300 lines/s, its maximum pulse repetition frequency is 2000 kHz, its angular measurement resolution is 0.001°, its scanning field of view is ±37.5°, and its laser divergence angle is 0.25 mrad. It has a distance measurement accuracy 0.02 m, a minimum measurement distance of 20 m, and a maximum flight altitude of 5600 m. The laser scanner operates at a 1000 m relative altitude with a spot size of 0.25 m and a scanning width of up to 1534 m. The scanner uses online waveform processing technology and has multiple echo recording ability. The LiDAR data used in this study are all LAS 1.2 point cloud data acquired from the processing of the CAF-LiTCHy integrated system flight study. The density of the point cloud is greater than 2.8 pts/m^2^, and the difference between point cloud bands is less than 0.1 m [28].

### 2.3. Data Preprocessing

#### 2.3.1. Measured Data Processing

The binary volume formula (*V* = a*D*^b^*H*^c^, *V* represents volume, *D* represents diameter at breast height, *H* represents tree height, and a, b, and c are estimated parameters) was used to estimate the stand volume based on the tree height and diameter at breast height of each tree in each standard plot. Due to different tree species, the selected binary volume formulas are also different (Table 1).

The standard plot volume can be obtained by the accumulation of each individual timber volume calculated by the parameters in Table 1. Combined with the area of each standard plot, the volume per hectare can be derived:

(1) $$M=\overset{N}{\underset{i=1}{\sum }}V_{i}/S$$

where *M* is the volume per hectare, the unit of which is m^3^/ha; *i* is number of stems; $V_{i}$ is the volume per tree; *S* is the area of standard sample plots.

#### 2.3.2. Airborne CCD Image Processing

In Agisoft PhotoScan Professional (Agisoft LLC, St. Petersburg, Russia), the structure from motion algorithm and the multi-view stereo algorithm are used to match the airborne CCD data with the same name, the dense matching point cloud is generated by the application of the least squares method to area network free parity and aerial triangulation, and the digital orthophoto map is generated by stitching together the orthophoto monoliths after constructing the grid and texture in Agisoft Photoscan Professional [28]. Based on the generated digital orthophoto map (DOM), the visible vegetation indices and texture features of each band in the study area were extracted separately via ENVI5.3 (Exelis VIS Company, Tysons Corner, VI, USA).

1. Extraction of visible vegetation index

With the help of the ENVI5.3 (Exelis VIS Company, Tysons Corner, VI, USA) software, waveband calculations were performed in the Band Math tool based on the DOM spectral information for the Mengjiagang forest area, and vegetation indices were calculated according to its formula. Considering the cost and the technology, only three bands of RGB (R: 647 nm; G: 553 nm; B: 461 nm) were acquired from the images, and the visible vegetation indices were extracted based on these three bands. The independent variables related to the band reflectance of Band 1, Band 2, and Band 3 were extracted, including the Normalized Green–Red Difference Index (NGRDI), the Extreme Green Index (EXG), the Color Index of Vegetation (CIVE), and another 17 independent variables (see Table 2).

2. Extraction of texture feature

Eight texture filters based on the second-order matrix are extracted from the DOM of the Mengjiagang forest region. These filters include mean, variance, homogeneity, contrast, heterogeneity, entropy, second moment, and correlation [26]. The second-order probability statistics use the gray level co-occurrence matrix to define and extract the relevant texture features’ values. Based on the single band of the remote sensing image, the final image data type is a 32-bit float of 8 channels, and the texture feature image is exported by a single channel. According to the gray level co-occurrence matrix calculated by this method, the gray level quantization level is 64, with a 3 × 3 processing window, and a transformation value of x, y = 1. The above eight texture features are calculated for the three bands of the image, and 24 texture feature variables are finally output.

3. Extraction of terrain factor

**Figure 2:**
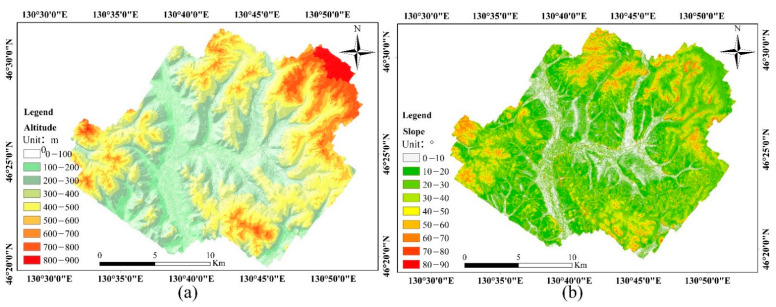
Distribution of altitude (**a**) and slope (**b**) in Mengjiagang forest area, extracted based on DEM data and GIS spatial analysis.

The digital elevation model (DEM) represents the bare-Earth surface, removing all natural and built features. The DEM is generated from the acquired airborne LiDAR data of the Mengjiagang forest area using triangulated irregular network (TIN) interpolation. In ArcGIS 10.7 (ESRI, Redlands, CA, USA), according to the standard plot coordinates, the altitude (h) and slope (slope) of each ground survey sample plot are extracted and mapped (Figure 2).

#### 2.3.3. Airborne LiDAR Data Processing

Airborne LiDAR data processing includes LiDAR waveform decomposition and geocoding, aerial declination, airband matching, point cloud pre-processing, point cloud rasterization processing, and a few other steps. The LiDAR data with geospatial information are generated using RiPROCESS (RIEGL, Horn, Austrian) laser processing software combined with POS information (based on the combination of Global Navigation Satellite System and Inertial Navigation System and receives the precise position and attitude information of each sensor when acquiring data) to encode the geographic location of the echo points of the LiDAR data. Four corner points and the center points of the sample plot are obtained via the RTK differential positioning method. 

In LiDAR 360 (Beijing Digital Green soil Technology Co., Ltd., Beijing, China), after pre-processing the acquired airborne LiDAR point cloud data, the sample area is cropped according to the sample area’s quadrangle points, and for each sample point cloud, we remove outliers, and ground point classification (filtering) and normalization (to remove the influence of terrain undulation on the elevation value of the point cloud data) are performed, etc. Ground point classification uses an improved progressive triangular irregular network (TIN) densification (IPDT) filtering algorithm, which firstly generates a sparse triangular network from the seed points and then condenses them layer by layer through iterative processing until all ground points are classified [52]. The normalized point cloud data are rasterized to produce a DEM and digital surface model (DSM). 

The Watershed algorithm is applied to the CHM generated from normalized point cloud samples, but the segmentation algorithm sometimes does not work very well. The results of single wood segmentation are examined in ALS editor, and due to the different densities of the sample plots, the effects of single wood segmentation are also different, with resulting undersegmentation, oversegmentation, or missing segmentation [53,54]. This necessitates the manual interactive editing of seed points, such as addition and deletion, to ensure that each tree in the sample plot has a seed point at the top. Finally, the accuracy of single wood segmentation is improved thanks to the edited seed points [55,56]. After the completion of individual wood segmentation in the sample plot, single tree parameters such as arithmetic mean height (H), mean crown width (W), and number of plants (N) are obtained for each standard plot. In addition, based on the normalized airborne LiDAR point cloud data, forest parameters such as the height and intensity of each ground sample plot are extracted. In this study, a total of 61 characteristic variables of two types have been extracted. The extraction results and characteristic variables of the LiDAR point cloud are shown in Table 3.

**Figure 3:**
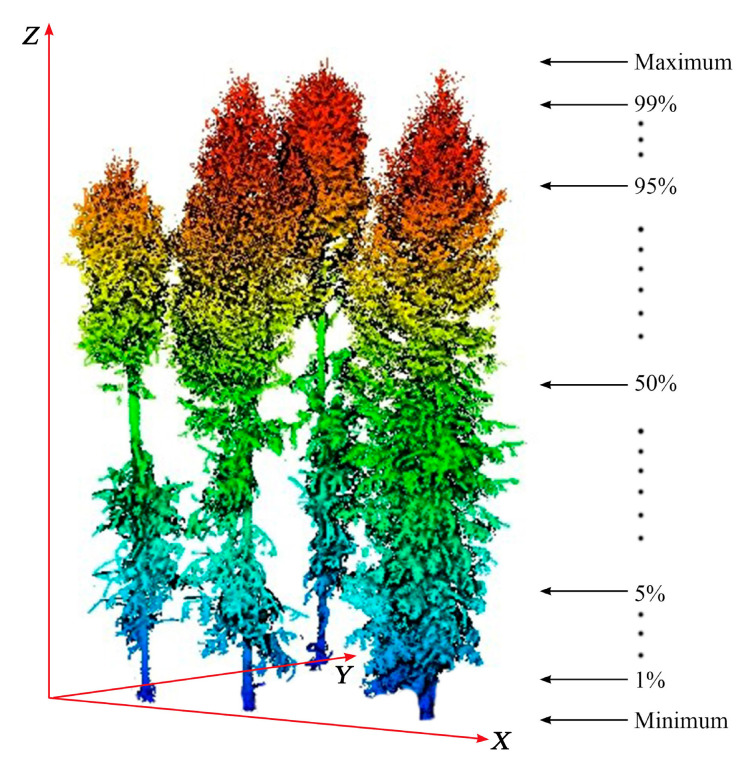
Diagram of point cloud feature variables. The point cloud space is divided into different grids, according to certain distances in the x and y directions, and then further divided into different “layers”, according to the specified height (z) interval.

The height variables are the parameters related to elevation and density and are calculated using the point cloud elevation values. The point cloud height percentile is the height percentile of a given statistical unit derived by ordering all the normalized LiDAR point clouds within it by height and then calculating the height at which X% of the points within each statistical unit are located. The maximum, minimum, standard deviation, median, and mean values are the maximum, minimum, standard deviation, median, and mean of the Z-values for all points within a given statistical unit, respectively. Skewness is the symmetry of the distribution of Z-values of all points within a certain statistical unit. The kurtosis is the flatness of the Z-value distribution of all points within a certain statistical unit. The coefficient of variation is the coefficient of variation of the Z-values of all points within a certain statistical unit. The density variables are divided into ten equal height slices from low to high, and the proportion of each layer of echoes to the whole echo is the corresponding density variable (Figure 3).

The intensity variable is similar to the height variable. The difference is that the intensity variable uses the intensity value of the point rather than the height value. Therefore, the intensity variables can be counted only when the point cloud data contain intensity information. The spatial point cloud is divided into different grids according to certain distances in the x and y directions; the intensity variables of each part are calculated using the point cloud intensity, and the interpretation of each intensity variable is referred to as the height variable.

### 2.4. Research Method

**Figure 4:**
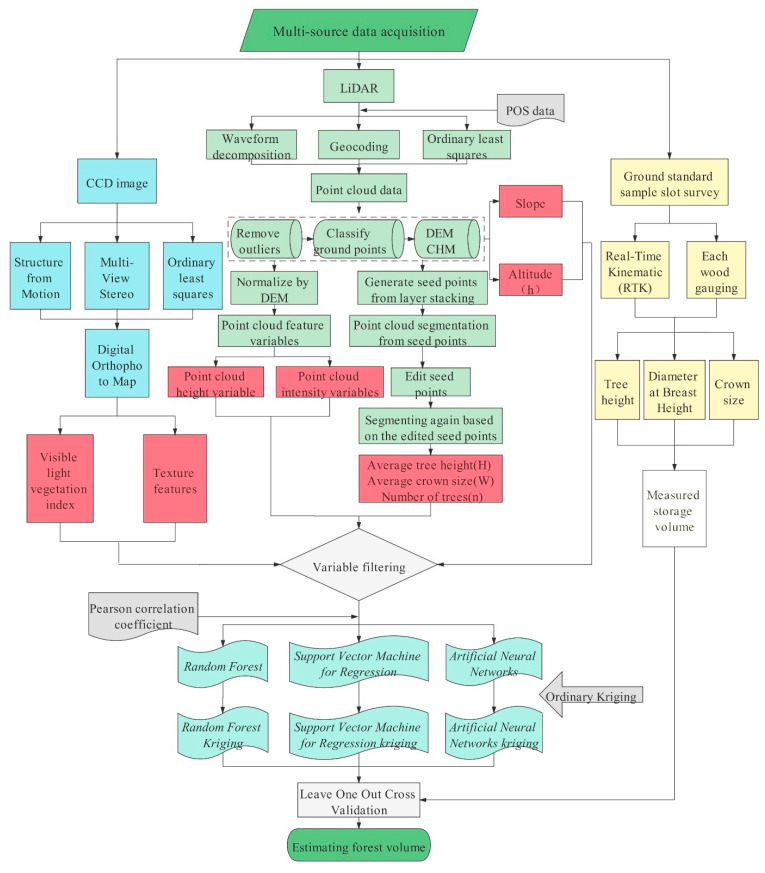
Technology roadmap. Multi-source data are used to estimate forest volume, where blue is the processing method of the CCD image data source; green is the processing method of the LiDAR data source; yellow is the processing method of the ground standard sample data source; red represents the independent variables; white on the left represents the measured value; blue–green represents the estimation model.

The workflow for estimating forest volume based on multi-source data is shown in Figure 4.

#### 2.4.1. Variable Screening

In this study, variables extracted based on LiDAR point cloud data include average tree height (H), average canopy width (W), and point cloud characteristic variables (see Table 3). The variables extracted from CCD images include the visible vegetation index (see Table 2) and the 24 texture features in three bands of RGB (8 texture features are extracted in each band). The variables extracted based on the digital elevation model include slope and elevation terrain factors. We finally add the areas of the standard plots, totaling 107 independent variables, with stand volume as the dependent variable. 

According to previous studies, it is not the case that the more independent variables used, the better [57]. Because there are more independent variables in this study, Pearson correlation analysis was applied before the model analysis. The correlation between these independent variables and the dependent variable volume was addressed, and a correlation coefficient significance test was carried out. The independent variables with large correlation coefficients and significant correlation were selected as candidate variables to participate in the model.

#### 2.4.2. Estimation Model of Forest Volume Based on Machine Learning

Using python scripting, after repeated experiments, suitable parameters were found using random search and grid search methods [58], and the inverse study area forest volume random forest (RF), support vector regression (SVR), and artificial neural networks (ANN) estimation models were established by combining the leave-one-out (Loo) cross-validation method with the coefficient of determination, the root mean square error, and the mean absolute error as model evaluation indexes.

1. RF model

**Figure 5:**
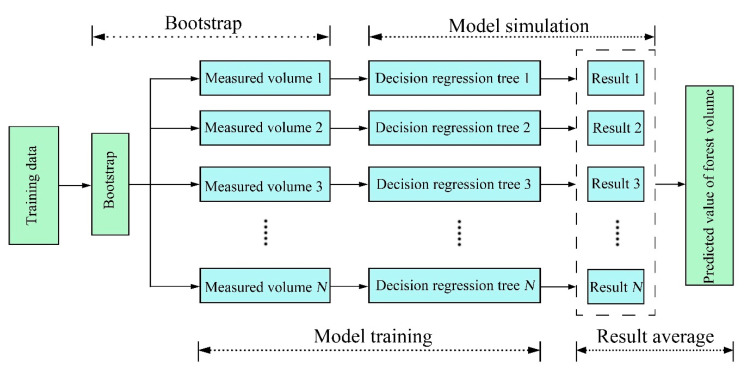
Schematic diagram of random forest regression. Through bootstrapping, a number of weak learners are trained by different decision regression trees, parameters, and features, and the final results are output by the weighted average method.

Random forest uses a random method to generate decision trees, which can be used for both classification and regression problems, while the random forest classifier can also handle missing values, and as long as there are enough trees, the classifier will not overfit the model (Figure 5) [24,39].

After repeated experiments, the maximum number of decisions of “n _ estimators” is set to 500, which is too small to be fitted, but any larger will increase the calculation quantity. Bootstrapping, whether there is a returned sample, is defaulted to Ture. The “max_features”, which is the maximum number of features to be considered when dividing, is set to 58 here. The “min _ sample _ split” is the minimum number of samples required for internal node repartition, and this value limits the conditions for subtrees to continue dividing. The value can be adjusted according to the sample size and is here set to 5. The “min _ sample _ leaf” leaf node has a minimum of 2 samples, depending on how many samples are adjusted. The “max_depth” decision maximum depth is set to 8, generally speaking, when there are fewer data or fewer features, regardless of this value. Here, as there are more feature variables, it is recommended to limit this maximum depth; the specific value depends on the distribution of data, but is usually between 10 and 100.

2. SVR model

Support vector machine (SVM) is a supervised learning method for classification, regression, and outlier detection. When the SVM is applied to regression fitting analysis, the basic idea is no longer to find an optimal classification surface to separate the two types of samples, but to find an optimal classification surface to minimize the error of all training samples. In order to use SVM for regression fitting, Vapnik et al. introduced the insensitive loss function on the basis of SVM classification and, thus, obtained support vector regression (SVR) [59,60]. The SVR function when used for volume (*M*) estimation can be defined as:

(2) $$M=\overset{N}{\underset{i=1}{\sum }}\left({\hat{a}}_{i}-a_{i}\right)k\left(x_{i},x\right)\;+\;b$$

where x is the vector of input predictor variables; $k\left(x_{i},x\right)$ is the kernel function; b is a constant; ${\hat{a}}_{i}$ and $a_{i}$ are weights (Lagrange multipliers). The constraint is Equation (3):

(3) $$\left\{\begin{array}{l} \sum_{i=1}^{N}\left({\hat{a}}_{i}-a_{i}\right)\;=0\\ 0\leq a_{i},{\hat{a}}_{i}\leq C \end{array}\right.$$

where *C* is the regularization parameter used to balance the training error and model complexity. The sequential minimum optimization algorithm is used to gradually solve the quadratic programming optimization problem, and the *M* equation is updated to map the new value until the Lagrange multiplier converges.

The radial basis kernel function is a kind of kernel function with strong locality, and it can, thus, map a sample into a higher dimensional space. This is one of the most widely used kernel functions. Both large and small samples perform well with this function, and it demands fewer parameters than polynomial kernel functions. Therefore, in most cases, the radial basis kernel function is preferred when one does not know which kernel function is used. It shows superior performance and robust results and is used in this study [61]:

(4) $$K\left(x_{i},x\right)=exp\left[\frac{{\left(x_{i}-x\right)}^{2}}{\sigma^{2}}\right]$$

The above equation involves the calculation of the Euclidean distance of two vectors; the radial basis kernel function is a monotonic function of the Euclidean distance of two vectors. σ is the bandwidth, which controls the radial range of effect; in other words, σ controls the local range of effect of the radial basis kernel function. When the Euclidean distance of $x_{i}$ and x is within a certain range, assuming a fixed $x_{i}$, $K\left(x_{i},x\right)$ changes significantly with x. In short, the training of the regression support vector model must find the best value of two meta-parameters: the regularization parameter (*C*) and kernel width (σ). Before debugging, the parameters must be used to normalize the data, choosing the radial basis kernel function (RBF) as the kernel function; the kernel function coefficient gamma is set to 0.001, while cross-validation is generally used to select the regularization parameter (*C*), here set to *C* = 150.

3. ANN model

**Figure 6:**
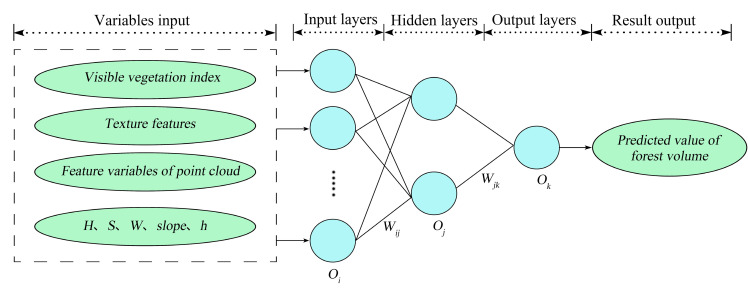
Structure model of artificial neural network. $O_{i}$ represents input neurals,$O_{j}$ represents hidden neurals. $O_{k}$ represents output neurals. Shown are the input neuron $O_{i}$, and each input to the hidden layer neuron $O_{j}$ is interconnected by selected weights. Then, the weighted output is combined and input into the output neuron $O_{k}$ to form the output value.

Artificial neural networks, also called neural networks, are a mathematical model formed using the working principles of biological neural networks (Figure 6). The neural network is one of many machine learning algorithms. It can be used for supervised tasks, such as classification and visual recognition, as well as unsupervised tasks. At the same time, it can deal with complex nonlinear problems, as its basic structure is neurons. A complete neural network consists of three parts: the input layer, hidden layer, and output layer [62,63].

Each node in the neural network accepts the input value and transmits this to the next layer. The input node directly transmits the input attribute value to the next layer (hidden or output layer). In a neural network, there is a functional relationship between the inputs and outputs of the nodes in the hidden and output layers, and this function is called the excitation function. The role of the excitation function in a neural network is, in layman’s terms, the transformation of multiple linear inputs into a nonlinear relationship. The rectified liner unit (relu, Equation (5)) function has the advantages of simple computation, simple derivatives, and fast convergence compared with other activation functions. The disadvantage is that the network is fragile, and it is easy for many neurons to have a value of 0 and, thus, never be trained again during training. However, this can be avoided by setting a suitable learning rate.

(5) $$\;Relu=\max\left(x,0\right)$$

Here, the artificial neural network with a 3-layer topology is chosen, i.e., 2 hidden layers and 1 output layer; the number of nodes in the first hidden layer is 50, while the number in the second hidden layer is also 50, and the number in the output layer is 1. The activation function from the first hidden layer to the second hidden layer is relu, and the activation function from the hidden layer to the output layer is also relu. The optimization algorithm selects the Adam function, and the learning rate is set to 0.01, the maximum number of learning epochs is set to 1000, and the tolerance is set to 20. Finally, the results of forest volume estimation in the study area are output.

#### 2.4.3. Estimation Model of Forest Volume Based on Ordinary Kriging Hybrid Method

Machine learning volume estimation models do not take into account the spatial autocorrelation of forest volume at small scales, and some studies have shown that there is some spatial autocorrelation in the errors of the above models. As such, a hybrid method considering both relevant environmental factors and spatial autocorrelation is generated, and this has higher prediction accuracy than a single model, such as the random forest Kriging (RFK) [64], regression support vector machine Kriging (SVRK) [57], and artificial neural network Kriging (ANNK) [65] models. At present, such hybrid methods are mostly used for soil attribute interpolation [66,67] and meteorological element interpolation [68,69], and their application in forest volume prediction model construction is rarely mentioned.

The RFK, SVRK, and ANNK models in this study used geostatistical analysis to perform ordinary Kriging interpolation on the residual parts of RF, SVR, and ANN prediction models. The results of the hybrid model are combined with the forest volume value $\hat{M}\left(a_{i},b_{i}\right)$ obtained by the regression of prediction factors and the results ($\bar{E}\left(a_{i},b_{i}\right)$) of the ordinary Kriging interpolation of regression residuals. The expressions are as follows:

(6) $$M\left(a_{i},b_{i}\right)=\hat{M}\left(a_{i},b_{i}\right)+\hat{E}\left(a_{i},b_{i}\right)$$

where $\left(a_{i},b_{i}\right)$ is the coordinate position of the predicted point; $\hat{M}\left(a_{i},b_{i}\right)$ is the prediction of stand volume based on RF, SVR, and ANN; $\hat{E}\left(a_{i},b_{i}\right)$ is the residuals of volume estimated from the RF, SVR, and ANN prediction; ordinary Kriging interpolation is an unbiased linear interpolation method. The expressions are as follows:

(7) $$\hat{E}\left(a_{i},b_{i}\right)=\overset{n}{\underset{i=1}{\sum }}{\hat{\lambda }}_{i}\hat{\beta }a_{i}b_{i}$$

where ${\hat{\lambda }}_{i}$ is the weight of the contributions of neighboring observations to the observation at point *i*, which can be determined by the semi-variance function. The semi-variance function, also known as the semi-variance moment, is a unique function of geostatistical analysis. Half of the variance of the difference between the values $Z\left(x\right)$ and $Z\left(x+h\right)$ of the regionalized variable $Z\left(x\right)$ at the points *x* and x+h is called the variance function of the regionalized variable $Z\left(x\right)$, denoted as $Y\left(h\right)$, and $2Y\left(h\right)$ is called the variance function. The expressions are as follows:

(8) $$Y\left(h\right)=\frac{1}{2N\left(h\right)}\overset{N\left(h\right)}{\underset{i=1}{\sum }}{\left[Z\left(x_{i}\right)-Z\left(x_{i}+h\right)\right]}^{2}$$

where $Y\left(h\right)$ is a semi-variance function; *h* is lag size; *N* (*h*) is the number of observations in the range *h*; $Z\left(x_{i}\right)$ is the observed value at $a_{i}$; $Z\left(x_{i}+h\right)$ is the observed value at $x_{i}+h$.

**Figure 7:**
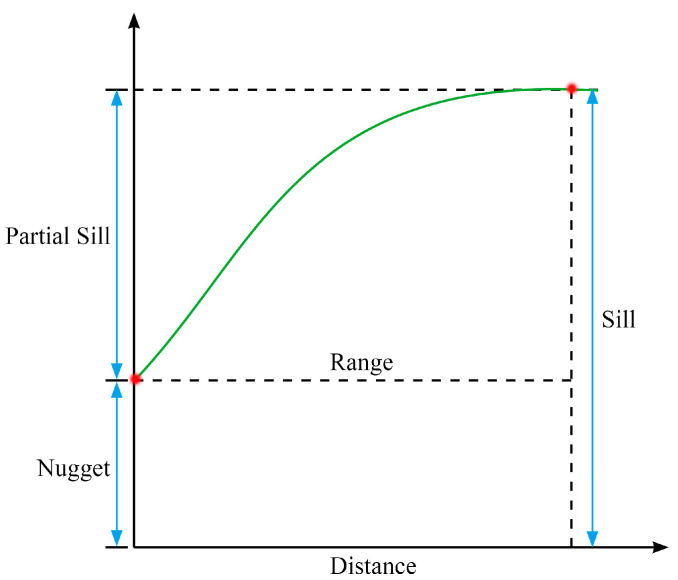
Semi-variance function theoretical diagram. Nugget represents the variation caused by measurement or scale. Sill represents the sum of random variation and fixed variation. Partial Sill is the difference between Sill and Nugget. When the value of the semi-variance function is taken from the initial Nugget to the Sill, the interval distance of the sampling points is called the Range. The Sill effect (Nugget/Sill) is an important indicator of the degree of spatial autocorrelation; the smaller its value, the stronger the degree of spatial autocorrelation.

The semi-variance function is a unique function in geostatistical analysis [57]. The semi-variogram is a set of discrete points and is often fitted into a mathematical model to represent the spatial autocorrelation of the measured samples. Spherical and Gaussian function models are used in our study. There are two very important points (red points) in the variation curve of this function (Figure 7): the point when the interval distance is 0 and the inflection point at which the semi-variance function tends to be smooth. From these two points, four corresponding parameters are generated: Nugget, Range, Sill, and Partial Sill. 

### 2.5. Model Evaluation

**Figure 8:**
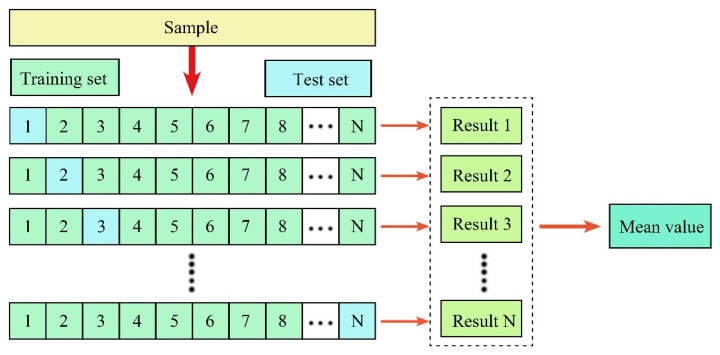
Diagram of leave-one-out cross-validation. There are N samples; each sample is used as a test sample, and the other N-1 samples are used as training samples. This yields N classifiers and N test results, and the average of these N results is used to measure the performance of the model.

Using python scripting, the prediction accuracy of the model is evaluated, with the $R_{Loo}^{2}$, $RMSE_{Loo}$, and $MAE_{Loo}$ obtained by the leave-one-out (Loo, Figure 8) method. The closer $R_{Loo}^{2}$ is to 1, the smaller the $RMSE_{Loo}$ and $MAE_{Loo}$ are, indicating that the model predicts more effectively. The leave-one-out method is used for evaluating learners in machine learning and is a special type of cross-validation [70]. The expressions of each evaluation index are as follows:

(9) $$R_{Loo}^{2}=\frac{1}{k}\overset{k}{\underset{j=1}{\sum }}R_{j}^{2}=\frac{1}{k}\overset{k}{\underset{j=1}{\sum }}\left(1-\overset{n_{j}}{\underset{j=1}{\sum }}{\left(O_{ij}-P_{ij}\right)}^{2}/\overset{n_{j}}{\underset{i=1}{\sum }}{\left(O_{ij}-{\bar{O}}_{j}\right)}^{2}\right)$$

(10) $$RMSE_{Loo}=\frac{1}{k}\overset{k}{\underset{j=1}{\sum }}RMSE_{j}=\frac{1}{k}\overset{k}{\underset{j=1}{\sum }}\sqrt{\frac{1}{n_{j}}\overset{n_{j}}{\underset{i=1}{\sum }}{\left(O_{ij}-P_{ij}\right)}^{2}}$$

(11) $$MAE_{Loo}=\frac{1}{k}\overset{k}{\underset{j=1}{\sum }}MAE_{j}=\frac{1}{k}\overset{k}{\underset{j=1}{\sum }}\left(\frac{1}{n_{j}}\overset{n_{j}}{\underset{i=1}{\sum }}\left|O_{ij}-P_{ij}\right|\right)$$

where k is the fold of cross-validation, and here, k=N; $O_{ij}$ and $P_{ij}$ represent the observation and the predicted value of the model for the jth time, respectively. ${\bar{O}}_{j}\;$ represents the average of the jth observation; $n_{j}$ represents the number of samples for the jth time; $R_{j}^{2}$,$\;RMSE_{j},\;\mathrm{and}\;MAE_{j}$ represent the coefficient of determination, root mean square error, and mean absolute deviation of the jth, respectively.

## 3. Results

### 3.1. Determining the Variables

**Figure 9:**
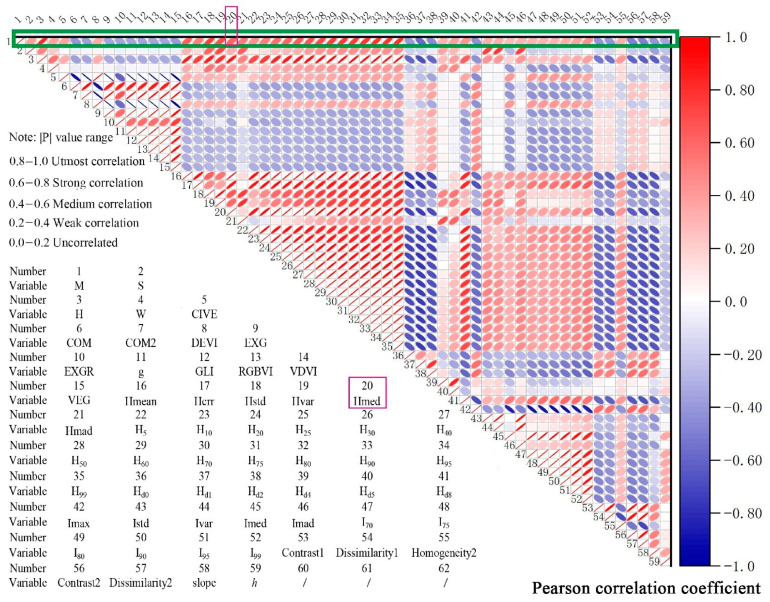
Plot of correlation coefficients between the dependent variable (volume M) and the independent variable. Red represents positive correlation and blue represents negative correlation, and the smaller the ellipse and the darker the color, the higher the correlation between the two variables. The green box indicates that the column is the correlation between the dependent variable and each independent variable, and each ordinal number in the figure represents a variable. For example, 20 represents Hmed, and its correlation with the dependent variable M(1) is the ellipse corresponding to the number 20 in the green box.

**Figure 10:**
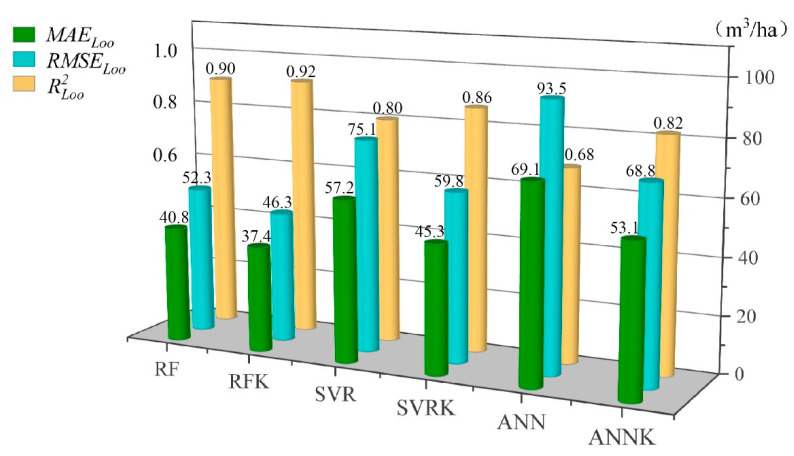
Model estimation evaluation indicators. The bar chart is a visual analysis of the data in Table 5; the three colors indicate the three indicators $MAE_{Loo}$, $RMSE_{Loo}$, and $R_{Loo}^{2}$. The models are analyzed by comparing the heights of the bars in the chart.

All the extracted variables were screened based on Pearson correlation analysis, and the screening results are shown in the correlation coefficient plot in Figure 9. In order to take into account all point cloud feature variables, visible vegetation indices, texture features, and topographic factors, variables with absolute values of correlation coefficients greater than or equal to 0.4 and *p*-values greater than 0.05 were selected, totaling 58 (all variables except volume M are explained in the lower left corner of Figure 10). Using these 58 variables as independent variables and the measured stand volumes as dependent variables, the RF, SVR, and ANN models were constructed in python. The RFK, SVRK, and ANNK hybrid models with ordinary Kriging interpolation were also constructed based on the residuals of the predicted values of these three models.

### 3.2. Comparison of Model Estimation Accuracy

According to the above research methods, RF, SVR, and ANN estimation models were established for different machine learning methods by inputting suitable parameters.

The above FR, SVR, and ANN models are used to estimate the volume of measured points (ground sample plots) in the study area, and the residual values based on these three models are obtained for OK interpolation. The parameters of the residual OK model are shown in Table 4. It can be seen from Table 4 that the three models’ residuals have a certain degree of spatial autocorrelation. Among these, as suggested by the Sill effect, the spatial autocorrelation of the SVR model residual is the strongest, and that of the RF model residual is the weakest. From the Range parameters, we infer that the spatial field fluctuation of the SVR model residual is the largest, and that of the RF model residual is the smallest. From the Nugget, we see that the randomness of the RF model residual is the smallest, and that of the ANN model residual is the largest. The estimated values of the RFK, SVRK, and ANNK hybrid models based on multi-source data forest accumulation can be obtained by adding the estimation values of RF, SVR, and ANN to the residual ordinary Kriging interpolation model.

For the RF, SVR, and ANN models and the RFK, SVRK, and ANNK hybrid models, the leave-one-out cross-validation method was used to validate the estimation accuracy and perform comparative analysis. The accuracy verification indexes of the models are shown in Table 5. It can be seen from Table 5 that the $RMSE_{Loo}$ and $MAE_{Loo}$ values of the RF model are the lowest, and the $R_{Loo}^{2}$ value is the highest, indicating that the error of the RF model is the smallest. The $RMSE_{Loo}$ and $MAE_{Loo}$ values of the ANN model are the highest, and the $R_{Loo}^{2}$ value is the lowest, indicating that the error of the artificial neural network model is the largest. Therefore, the best-performing model of these three machine learning methods is achieved by the RF model, followed by the SVR model, and the worst-performing model is achieved by ANN.

By contrast, the estimation accuracy of the hybrid models is improved to different degrees, i.e., combining the ordinary Kriging model with the residuals of the machine learning model can greatly reduce the error in the estimation of forest volume. The $R_{LOO}^{2}$ of the estimation accuracy of these six models is above 0.6; the ANNK model undergoes the largest improvement in estimation accuracy, and the RFK model shows the smallest improvement in estimation accuracy. However, the RF and RFK models’ $R_{LOO}^{2}$ value is above 0.9, while their $RMSE_{Loo}$ and $MAE_{Loo}$ values are the smallest; the model’s estimation accuracy is much higher than the other models, and the estimation effect is the best. Therefore, the hybrid model RFK is the optimal model for forest volume estimation based on the multi-source data used in this study.

The level of accuracy improvement is the percentage improvement in the root mean square error of the hybrid method compared with the machine learning model. For example, the level of accuracy improvement = [$RMSE_{Loo}$(RF) − $RMSE_{Loo}$(RFK)]/$RMSE_{Loo}$(RF).

## 4. Discussion

### 4.1. Multi-Source Data

Forest volume refers to the total amount of tree volume in a certain forest area, which is one of the basic indicators reflecting the overall scale and level of forest resources in a country or region. It is closely related to wood safety, climate change, animal habitat, etc., and can provide a scientific basis for forest management plans. The accurate estimation of forest volume is of great significance for improving the management level of forest resources and ecological environmental protection [34].

The multi-source data used in this study are CCD remote sensing images, LiDAR data, and the actual measurement data from ground standard sample plots. In the field survey, we investigated the data of three standard sample plots of different tree species used for forest volume estimation. Differently from other studies, the CCD remote sensing images and LiDAR data obtained from the study area were accessed with the same equipment, CAF-LiTCHy, and share the same positioning system, which avoids the errors caused by the inaccurate matching of the two data sources and improves the accuracy of forest volume estimation. The CCD remote sensing images and LiDAR data were obtained using the CAF-LiTCHy integrated system at different times, but some studies have shown that the interannual variation in the vegetation index is small. For this paper, the tree species assessed were primarily *Larix olgensis*, *Pinus koraiensis*, and *Pinus sylvestris*. These coniferous species grow slowly, and the interannual variation in their surface characteristics is not large. Therefore, the research results will be less affected by time [71].

We here extracted the visible vegetation index, and although this is less sensitive to vegetation than the index extracted using the near infrared band, other scholars have also studied visible vegetation indexes, such as the visible-band different vegetation index (VDVI) instead of the NDVI [22,72]. These remote sensing factors have great significance in machine learning and local regression estimation. Therefore, the CCD remote sensing variables used in this study can be used as auxiliary variables for the estimation of forest volume. At the same time, texture feature parameters will be affected by the different sizes of the extraction window, and terrain factor will also have a negative impact on the estimation of forest volume due to the terrain shadow in the remote sensing image. In addition, the spectral characteristics of remote sensing images may be limited by saturation. In a certain area, two different ground objects may present the same spectral curve characteristics; it may also be the same ground object, in different growth states, showing different spectral line characteristics. The point cloud height variable of LiDAR data can effectively reflect such information as the average height of the forest, and the point cloud intensity variable also reflects the density and horizontal structure of the forest to a certain extent. Combining these two important variables, it is possible for airborne LiDAR to estimate forest volume. In order to avoid the deficiencies of using optical remote sensing data for estimating forest volume, our study introduced the point cloud characteristic variables of LiDAR point cloud data and formed a variety of data sources to jointly construct a forest volume estimation model.

The data used in this study do not consider the effects of undergrowth herbs, shrubs, litter, and soil the environment on forest volume, which is an area in which remote sensing technology also struggles. If these variables can be incorporated into the study of forest volume, the accuracy will be greatly improved.

### 4.2. RF, SVR, and ANN

Before building a forest volume estimation model, all the variables obtained were screened for Pearson correlation coefficients; however, the screening results still retained many variables, so the conventional parametric model definitely did not work well. In order to overcome the shortcomings of using traditional statistical methods in forest growth and harvest research, RF, SVR, and ANN were used to estimate the forest volume in the study area, with good results [40]. It can be seen from Figure 10 that among the three machine learning methods, the test index of the RF estimation model is the best, with the best effect on the estimation of forest volume. The reason for the RF model’s superiority is that, compared with other machine algorithms, the random forest algorithm does not need to repeatedly adjust its parameters, and it can handle high-latitude data (data with many characteristic variables). Even if some characteristic variables are missing, it can maintain accuracy in its final results. It is less affected by collinearity and outliers and has strong generalizability, so it is not easy to overfit. The SVR and ANN algorithms are vulnerable to collinearity when there are many independent variables—especially the ANN algorithm. There are too many uncertainties in this mode of estimation, such as learning efficiency, the number of hidden layer nodes, the selection of training functions, etc. Because of the hidden black box operation of this method, the relationship between input and output cannot be accurately expressed and analyzed [40,61,72]. Since many independent variables are used in the inversion of forest volume in this study, some variables cannot avoid collinearity. Combining the advantages and disadvantages of the above three algorithms, the RF machine learning algorithm is more suitable for the inversion of forest volume, and its estimation effect is the best.

**Figure 11:**
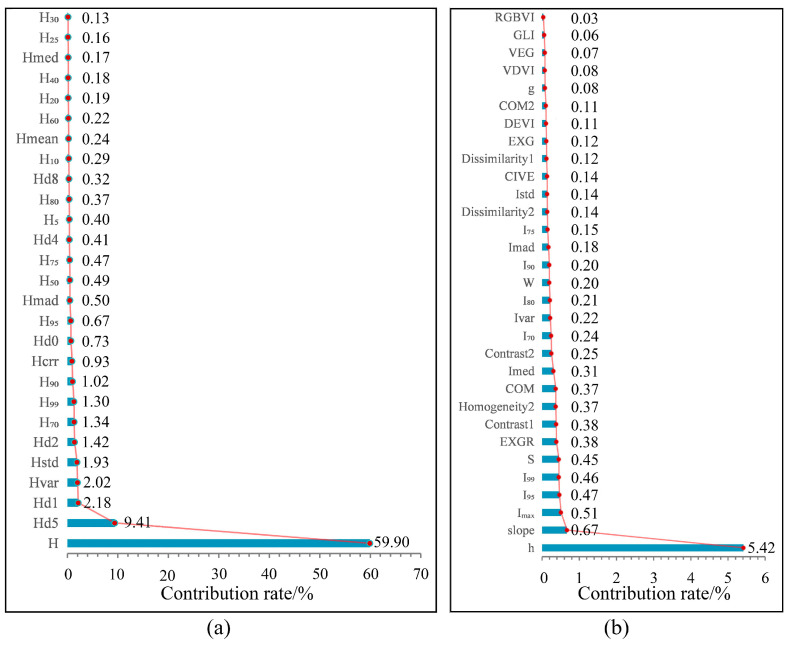
Importance of random forest characteristic variables. Using a python script, the importance analysis of all variables involved in the construction of the forest volume estimation model was performed in the random forest algorithm; each variable was scored in order of its contributory magnitude and (**a**) shows the score ranking of variables associated with the height of point cloud, while (**b**) shows the score ranking of other variables.

In addition, the RF algorithm can rank the importance scores of feature variables and analyze the contributions of variables to model prediction. The higher the score, the greater the contribution to model estimation. As can be seen from Figure 11, among the height variables with a high correlation with volume, H, H_d5_, H_d1_, H_var_, H_std_, H_70_, and H_99_ had high importance scores and contributed significantly to model estimation. Tree height and the volume of accumulation are closely related, and a variety of models have been developed for tree height storage in many studies [73,74,75]. Among the other feature variables, the topographic factors of slope and altitude (h) contribute the most to modeling, followed by the presence of point cloud variables with over 90% intensity, EXGR, the COM visible fingerprint index, and the texture features of contrast in Band 1 and homogeneity in Band 2. There is greater human activity in woodlands with a gentle slope and low altitude, which is also conducive to forest harvesting; where forests are vulnerable to destruction, the forest volume is low. With the increase in altitude, the slope becomes steep, and human activities are reduced, which is not conducive to forest harvesting, meaning the forest site quality is high, and the forest volume is high. Using the visible vegetation index, texture features and terrain factors as auxiliary variables in forest volume model estimation can reduce the error to a certain extent; these are important remote sensing factors that cannot be ignored.

### 4.3. RFK, SVRK, and ANNK

**Figure 12:**
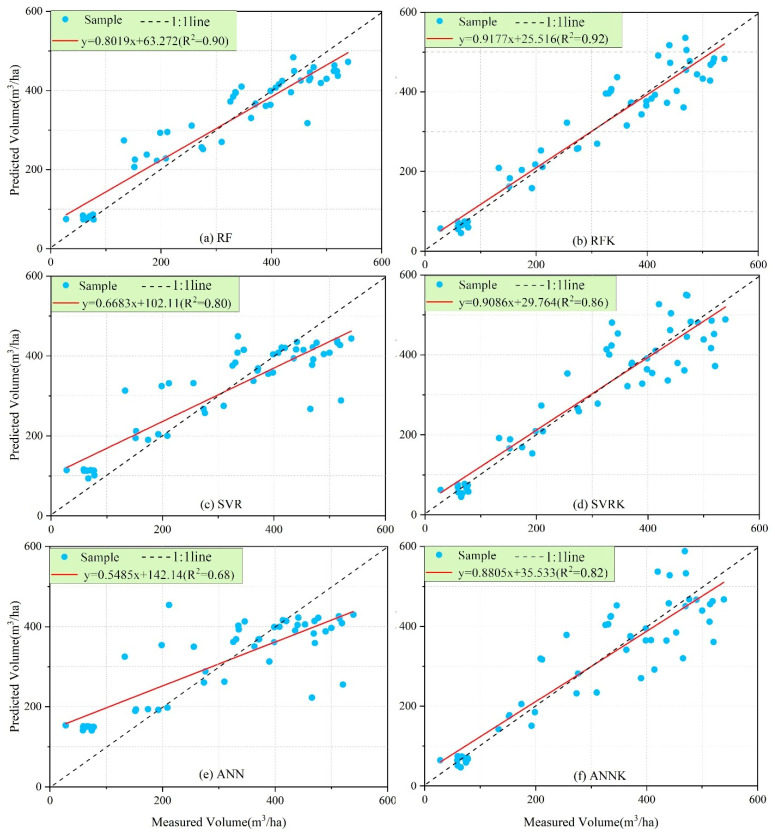
Scatter plot of the estimated and measured values of the six models ($R^{2}\;$=$\;R_{LOO}^{2}$). The above six models were compared in order to analyze the accuracy of model predictions by establishing a linear relationship between measured and predicted volume values.

The RFK, SVRK, and ANNK hybrid models are extensions of the RF, SVR, and ANN models, which take into account the inherent spatial correlation structure of the residuals of the RF, SVR, and ANN models and perform ordinary Kriging interpolation on the residual part of the fit of the RF, SVR, and ANN models. They then sum the residual part with the trend term of the fit of the RF, SVR, and ANN models and perform a test, which improves the estimation accuracy [57,64,65]. Figure 10 and Figure 12 show that the estimation models incorporating the geostatistical analysis semi-variance function method have an improved estimation accuracy compared with the original models. The estimation accuracy of the ANNK hybrid model is the most improved compared with the original model; its $R_{LOO\;}^{2}$ increased by 0.137 and its $RMSE_{Loo}$ accuracy increased by 26.24%. This is followed by the SVRK and RFK hybrid models. Overall, the forest volume estimation effect of the RF model and RFK hybrid model based on multi-source data is the best. The RFK hybrid model’s estimation effect is better than that of the RF model, as the estimated value and measured value of the 1:1 scatter plot fitting effect are the best.

## 5. Conclusions

With the development of remote sensing technology in the forestry industry, it has become easier to obtain data on a large number of variables, while traditional statistical research methods can no longer keep up with this trend. To achieve the goal of estimating forest volume scientifically and efficiently, this study uses a machine learning method with a strong generalization ability and high efficiency to construct RF, SVR, and ANN models to estimate forest volume based on multi-source data, and the RF model has the best prediction effect. At the same time, considering the spatial correlation of sample sites, a hybrid model of RFK, SVRK, and ANNK was constructed to improve the accuracy of forest volume estimation, and the RFK model has the best prediction effect. We hope that the research method proposed in this study can not only collect timely and accurate data (especially LiDAR data) for forest resource management, but also provide accurate and efficient data analysis methods, so as to provide scientific and technical support for the sustainable development of forest management. We have come to the following conclusions:

(1) The machine learning method has a good effect on the estimation of multivariable forest volume, but the shortcomings of machine learning are that it ignores the spatial autocorrelation of neighboring observed data.
(2) By using machine learning to estimate stock volume, the accuracy has been improved to varying degrees when considering the spatial correlation effect. RF is optimal compared with other machine learning methods due to its specific advantages, making it also the optimal basic estimation model for the mixed model. The RFK estimation model is the best among the six models (Figure 10).
(3) As can be seen from Figure 12, among the variables involved in model construction, the variables extracted from LiDAR data are much greater in number than those extracted from other data sources, and their importance scores are also relatively high. This shows that LiDAR data can express forest volume more accurately and provide an accurate and efficient method for future forest resources investigation.
(4) Forest resources are amongst the most important resources on Earth, and they play an irreplaceable role in carbon sequestration, slowing down global warming and maintaining biodiversity. We have studied them in order to better protect and use them and are striving to achieve the sustainable development of the environment.

## Figures and Tables

**Table 1:** The binary volume table parameters of main coniferous forest species in Northeast China [48].

Tree Species	a	b	c
*Larix olgensis*	0.00005017	1.7583	1.14967
*Pinus koraiensis*	0.00006353	1.9436	0.89689
*Pinus sylvestris*	0.00006938	1.7631	1.03701

Note: a, b, and c are the parameter estimates.

**Table 2:** Extracted visible light vegetation index [22,23,49,50,51].

Vegetation Index	Abbreviation	Calculation Formula
Normalized Green–Red Difference Index	NGRDI	(G − R)/(G + R)
Extreme Green Index	EXG	2g − r − b
Color Index of Vegetation	CIVE	0.44r − 0.88g + 0.39b + 18.79
Vegetation Index	VEG	g/r^a^b^1−a^, a = 0.67
Excess Green Minus Excess Red Index	EXGR	EXG − 1.4r − g
Woebbecke Index	WI	(g − b)/(r − g)
Visible Band Different Vegetation Index	VDVI	(2G − R − B)/(2G + R + B)
Red–Green Ratio Index	RGRI	r/g
Normalized Green–Blue Difference Index	NGBDI	(G − B)/(G + B)
Green–Blue Ratio Index	GBRI	b/g
Green–Red and Blue Vegetation index	GBRVI	(G^2^ − B × R)/(G^2^ + B × R)
Modified Green and Red Vegetation Index	MGRVI	(G^2^ − R^2^)/(G^2^ + R^2^)
Differential Enhanced Vegetation Index	DEVI	G/3G + R/3G + B/3G
Green Leaf Index	GLI	(2g − r − b)/(2g + r + b)
Combination Index	COM	0.25EXG + 0.3EXGR + 0.33CIVE + 0.12VEG
Combination Index 2	COM2	0.36EXG + 0.47CIVE + 0.17VEG
Excess Red Index	EXR	1.4 × r − g

R: red light channel. G: green light channel. B: blue light channel. r: standardized results for the red light channel, r = R/(R + G + B). g: standardized results for the green light channel, g = G/(R + G + B). b: standardized results for blue light channels, b = B/(R + G + B).

**Table 3:** Extracted point cloud characteristic variables [30].

Point Cloud Characteristic Variable		Description
Point cloud height variable	H_1_, H_5_, H_10_, H_20_, H_25_, H_30_, H_40_, H_50_, H_60_, H_70_, H_75_, H_80_, H_90_, H_95_, H_99_	Point cloud height percentile
	H_max_, H_min_, H_mean_, H_med_, H_std_, H_var_, H_mad_	Maximum, minimum, average, median, standard deviation, variance, and mean absolute deviation of point cloud height
	H_skew_, H_kurt_, H_crr_, H_cv_	Skewness, kurtosis, canopy fluctuation rate, and coefficient of variation of point cloud height
	H_d0_, H_d1_, H_d2_, H_d3_, H_d4_, H_d5_, H_d6_, H_d7_, H_d8_, H_d9_	Point cloud height density variable
Point cloud intensity variable	I_1_, I_5_, I_10_, I_20_, I_25_, I_30_, I_40_, I_50_, I_60_, I_70_, I_75_, I_80_, I_90_, I_95_, I_99_	Point cloud intensity percentile
	I_max_, I_min_, I_mean_, I_med_, I_std_, I_var_, I_mad_	Maximum, minimum, average, median, standard deviation, variance, mean absolute deviation of point cloud intensity
	I_skew_, I_kurt_, I_cv_	Skewness, kurtosis, and coefficient of variation of point cloud intensity

**Table 4:** Parameters of ordinary Kriging models of residuals and their accuracy.

Residual (m^3^/ha)	Model	Range (km)	Nugget	Partial Sill	Sill Effect (Nugget/Sill)	*MAE* (m^3^/ha)	*RMSE* (m^3^/ha)	*R* ^2^
R_RF_	Spherical	3.05	803.01	1772.39	0.31	43.9	46.3	0.25
R_SVR_	Gaussian	4.001	1136.22	4560.93	0.20	59.8	53.4	0.40
R_ANN_	Gaussian	3.572	1975.30	4668.24	0.30	65.3	68.8	0.49

**Table 5:** Estimation accuracy evaluation of machine learning models and ordinary Kriging model hybrid.

Model	$MAE_{Loo}\;(m^{3}/\mathrm{ha})$	$RMSE_{Loo}\;(m^{3}/\mathrm{ha})$	$R_{LOO}^{2}$	$R_{LOO\;}^{2}\mathrm{Accuracy}\;\mathrm{Improvement}$	Level of Accuracy Improvement (%)
RF	40.8	52.3	0.90	/	/
SVR	57.2	75.1	0.80	/	/
ANN	69.1	93.5	0.68	/	/
RFK	37.4	46.3	0.92	0.02	11.47%
SVRK	45.3	59.8	0.86	0.06	20.37%
ANNK	53.1	68.8	0.82	0.14	26.42%
